# Supplementary material for: Pyruvate Dehydrogenase Kinase 1 inhibition mediated oxidative phosphorylation enhancement in cartilage promotes osteoarthritis progression
Source: BMC Musculoskelet Disord. 2023 Jul 20;24:597. doi: 10.1186/s12891-023-06585-6 (PMC10357736; doi:10.1186/s12891-023-06585-6)
Supplement: Supplementary file 1 — Additional file 1. [file 12891_2023_6585_MOESM1_ESM.zip › BP.Gsea.1653623667859/GOBP_PROTEIN_DNA_COMPLEX_DISASSEMBLY.html]

Details for gene set GOBP\_PROTEIN\_DNA\_COMPLEX\_DISASSEMBLY[GSEA]

|  || Dataset | 55457\_collapsed\_to\_symbols.cls.cls #OA\_versus\_normal.cls.cls #OA\_versus\_normal\_repos |
| Phenotype | cls.cls#OA\_versus\_normal\_repos |
| Upregulated in class | normal |
| GeneSet | GOBP\_PROTEIN\_DNA\_COMPLEX\_DISASSEMBLY |
| Enrichment Score (ES) | -0.5627649 |
| Normalized Enrichment Score (NES) | -1.7276592 |
| Nominal p-value | 0.007561437 |
| FDR q-value | 0.11909851 |
| FWER p-Value | 1.0 |
Table: GSEA Results Summary

  

Fig 1: Enrichment plot: GOBP\_PROTEIN\_DNA\_COMPLEX\_DISASSEMBLY      
 Profile of the Running ES Score & Positions of GeneSet Members on the Rank Ordered List

  

| SYMBOL | TITLE | RANK IN GENE LIST | RANK METRIC SCORE | RUNNING ES | CORE ENRICHMENT || 1 | H2AW | H2A.W histone [Source:HGNC Symbol;Acc:HGNC:20507] | 4143 | 0.143 | -0.2986 | No |
| 2 | SMARCE1 | "SWI/SNF related, matrix associated, actin dependent regulator of chromatin, subfamily e, member 1 [Source:HGNC Symbol;Acc:HGNC:11109]" | 4255 | 0.132 | -0.2858 | No |
| 3 | SMARCD3 | "SWI/SNF related, matrix associated, actin dependent regulator of chromatin, subfamily d, member 3 [Source:HGNC Symbol;Acc:HGNC:11108]" | 5036 | 0.070 | -0.3350 | No |
| 4 | SMARCD2 | "SWI/SNF related, matrix associated, actin dependent regulator of chromatin, subfamily d, member 2 [Source:HGNC Symbol;Acc:HGNC:11107]" | 5129 | 0.062 | -0.3322 | No |
| 5 | SUPT16H | "SPT16 homolog, facilitates chromatin remodeling subunit [Source:HGNC Symbol;Acc:HGNC:11465]" | 5174 | 0.058 | -0.3262 | No |
| 6 | ARID1A | AT-rich interaction domain 1A [Source:HGNC Symbol;Acc:HGNC:11110] | 5735 | 0.013 | -0.3677 | No |
| 7 | SMARCC1 | "SWI/SNF related, matrix associated, actin dependent regulator of chromatin subfamily c member 1 [Source:HGNC Symbol;Acc:HGNC:11104]" | 8052 | -0.159 | -0.5217 | No |
| 8 | SMARCC2 | "SWI/SNF related, matrix associated, actin dependent regulator of chromatin subfamily c member 2 [Source:HGNC Symbol;Acc:HGNC:11105]" | 8480 | -0.192 | -0.5238 | Yes |
| 9 | SSRP1 | structure specific recognition protein 1 [Source:HGNC Symbol;Acc:HGNC:11327] | 8692 | -0.208 | -0.5065 | Yes |
| 10 | NFE2 | "nuclear factor, erythroid 2 [Source:HGNC Symbol;Acc:HGNC:7780]" | 9385 | -0.263 | -0.5177 | Yes |
| 11 | HMGA1 | high mobility group AT-hook 1 [Source:HGNC Symbol;Acc:HGNC:5010] | 9966 | -0.318 | -0.5114 | Yes |
| 12 | SMARCA4 | "SWI/SNF related, matrix associated, actin dependent regulator of chromatin, subfamily a, member 4 [Source:HGNC Symbol;Acc:HGNC:11100]" | 10009 | -0.322 | -0.4626 | Yes |
| 13 | SMARCB1 | "SWI/SNF related, matrix associated, actin dependent regulator of chromatin, subfamily b, member 1 [Source:HGNC Symbol;Acc:HGNC:11103]" | 10117 | -0.333 | -0.4171 | Yes |
| 14 | TNP1 | transition protein 1 [Source:HGNC Symbol;Acc:HGNC:11951] | 11165 | -0.453 | -0.4252 | Yes |
| 15 | GRWD1 | glutamate rich WD repeat containing 1 [Source:HGNC Symbol;Acc:HGNC:21270] | 11694 | -0.531 | -0.3804 | Yes |
| 16 | SMARCD1 | "SWI/SNF related, matrix associated, actin dependent regulator of chromatin, subfamily d, member 1 [Source:HGNC Symbol;Acc:HGNC:11106]" | 11991 | -0.592 | -0.3077 | Yes |
| 17 | SET | SET nuclear proto-oncogene [Source:HGNC Symbol;Acc:HGNC:10760] | 12000 | -0.593 | -0.2125 | Yes |
| 18 | RPL23 | ribosomal protein L23 [Source:HGNC Symbol;Acc:HGNC:10316] | 12558 | -0.777 | -0.1302 | Yes |
| 19 | MYC | "MYC proto-oncogene, bHLH transcription factor [Source:HGNC Symbol;Acc:HGNC:7553]" | 12801 | -0.970 | 0.0077 | Yes |
Table: GSEA details [plain text format]

  

Fig 2: GOBP\_PROTEIN\_DNA\_COMPLEX\_DISASSEMBLY      
 Blue-Pink O' Gram in the Space of the Analyzed GeneSet

  

Fig 3: GOBP\_PROTEIN\_DNA\_COMPLEX\_DISASSEMBLY: Random ES distribution      
 Gene set null distribution of ES for **GOBP\_PROTEIN\_DNA\_COMPLEX\_DISASSEMBLY**
